# Supplementary material for: Sound Localization Ability in Dogs
Source: Vet Sci. 2022 Nov 8;9(11):619. doi: 10.3390/vetsci9110619 (PMC9694642; doi:10.3390/vetsci9110619)
Supplement: Supplementary file 1 [file vetsci-09-00619-s001.zip › vetsci-1973651-supplementary.pdf]

Table S1: Test-related data of dogs.

Dog	Mean ± SD of the last six reversals in the ascending procedure	N° of sessions to reach the 1st of the last six reversals in descending (D) and ascending (A) procedures
1	5.2 ± 1.0°	D = 15; A = 5
2	11.2 ± 1.0°	D = 11; A = 13
3	1.3 ± 0.5°	D = 25; A = 11
4	6.2 ± 0.75°	D = 14; A = 9
5	6.5 ± 0.5°	D = 20; A = 6
6	6.3 ± 0.8°	D = 24; A = 9
7	5.8 ± 0.75°	D = 14; A = 12
8	6.2 ± 1.2°	D = 15; A = 24
9	13.2 ± 1.2°	D = 17; A = 11
10	11.5 ± 1.2°	D = 14; A = 10
